# Ptosis following botulinum toxin injection in hemifacial spasm

**Published:** 2019-10-07

**Authors:** Mansooreh Jamshidian-Tehrani, Hadi Z. Mehrjardi, Abolfazl Kasaee, Samira Yadegari

**Affiliations:** Eye Research Center, Farabi Eye Hospital, Tehran University of Medical Sciences, Tehran, Iran

**Keywords:** Hemifacial Spasm, Botulinum Toxins, Blepharoptosis, Aneurysm

Hemifacial spasm (HFS) is characterized by irregular involuntary tonic or clonic contractions of muscles innervated by the seventh cranial nerve. Patients usually need long-term treatment, as spontaneous remission is infrequent.

Repeated botulinum toxin injection has been shown as a safe and successful treatment for symptomatic relief in patients with HFS.^1^ Side effects are usually mild and transient. Ptosis has been reported in about 24% of patients with HFS probably due to diffusion of toxin to levator palpebrae superioris muscle.^2^ However, the frequency of ptosis in patients with HFS has not been addressed yet, due to causes other than botulinum toxin side effect.

Herein, we present a case of HFS who presented to our clinic with complaint of complete ptosis and progressive pain early after botulinum toxin injection. Despite initial negative evaluations, further work up revealed a compressive lesion.

A 75-year-old man presented with left severe ptosis since 3 weeks ago (Figure 1). He was known case of left HFS since 4 years ago, and botulinum toxin had been regularly injected for his symptom relief in orbicularis oculi, corrugator, and procerus muscles. 

**Figure 1:**
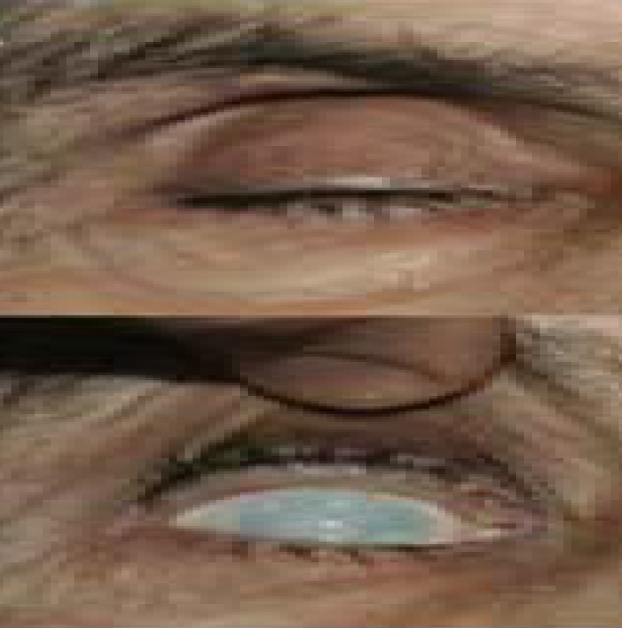
Left Blepharoptosis one week after botulinum toxin injection

The patient stated that this new ptosis had begun within a week after his last Dysport (Ipsen, Ltd., Slough, Berkshire, UK) injection while he had no ptosis in his previous injections. In past medical history, he had ischemic heart disease, hypertension, cataract extraction of both eyes, and glaucoma surgery on his right eye. He had been admitted to the neurology ward of a general hospital.

His brain magnetic resonance imaging (MRI) and MR angiography (MRA) in that hospital had shown dolichoectasia of distal segment of left cervical internal carotid artery and left vertebral and basilar arteries (Figure 2). The patient had been discharged with the diagnosis of "ptosis secondary to Dysport injection".

**Figure 2:**
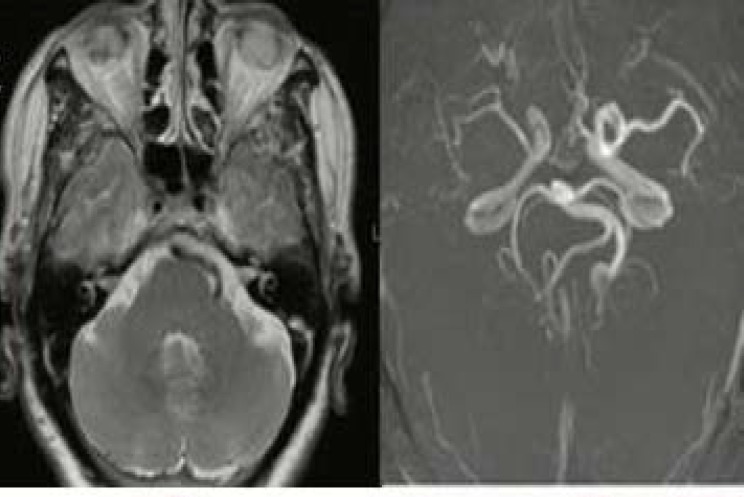
Magnetic resonance imaging (MRI) and angiography revealed dolichoectasia of distal left internal carotid artery and left vertebral and basilar arteries, but no apparent aneurysm

In our clinic, he had severe periorbital pain, which had made him restless. On examination, visual acuity was no light perception in right eye (due to a complicated cataract surgery and advanced glaucoma) and 7/10 in left eye. He had complete ptosis on the left side. With lifting ptotic lid, we observed severe limitation in adduction, depression, and elevation of left eye. His left pupil was irregular, and its reaction to light was sluggish. Other ocular exams including intraocular pressure and slit lamp assay were within normal limits.

Based on these findings, left complete third cranial nerve palsy was diagnosed for him with pupil that was non-reliable due to irregularity of old cataract surgery. Because of the severe disabling periorbital pain that was getting aggravated overtime, and was refractory to several intravenous types of opioids and consequently high clinical suspicion to aneurysm, a brain computed tomographic angiography (CTA) was scheduled for him. It revealed an aneurysm (7.2 × 6.6 mm with neck diameter of 1.6 mm) in left internal carotid artery just before posterior communicating artery (PCOM) branch (Figure 3). With the diagnosis of compressive third cranial nerve palsy, the patient underwent aneurysm clamping. Pain resolved within few days after the operation. One-month post operation, ptosis and ocular motility improved as well (Figure 4).

**Figure 3:**
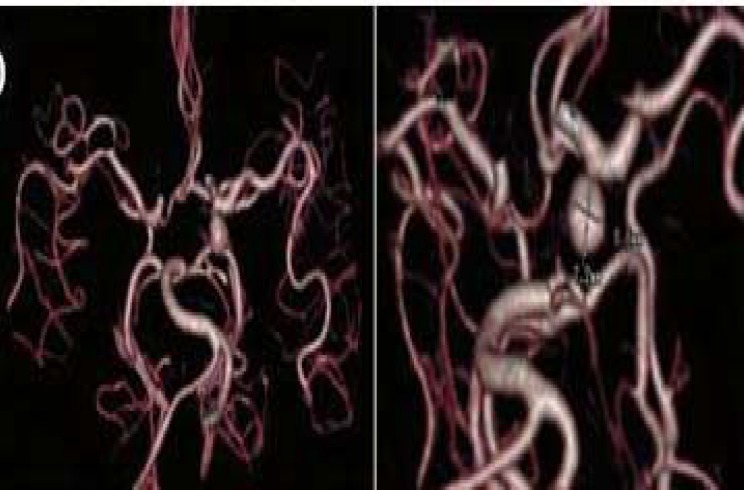
Brain computed tomography angiography revealed an aneurysm in left posterior communicating artery (PCOM)

Botulinum toxin has gained popularity in symptomatic management of patients with HFS despite the need for repeated injections and high costs.^3^^,^^4^ This approach is generally safe, provides quick response and possible side effects including blepharoptosis that is usually mild and transient. Ptosis attributed to botulinum injection usually manifests within 2-3 days after the injection, peaks at about 7 days and gradually improves within weeks. 

**Figure 4:**
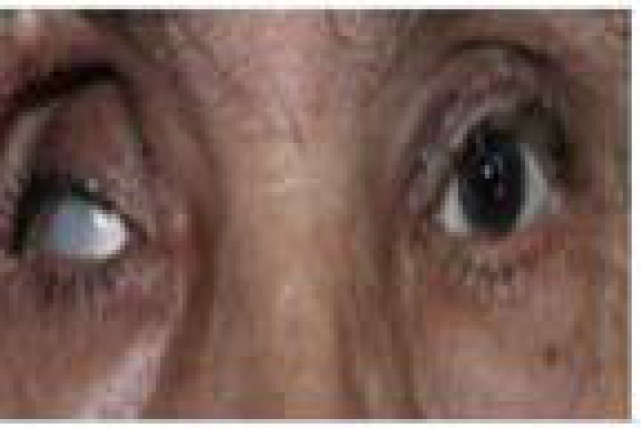
Blepharoptosis has been improved one-month post operation of aneurysm

In our patient, though ptosis developed one week after the injection, but was progressive and associated with severe aggravating pain. More importantly, detailed examination of ptotic eye revealed third cranial nerve palsy with irregular pupil. His brain MRI and MRA were unremarkable for a compressing lesion despite the presence of a PCOM aneurysm in the brain CTA. Therefore, MRA may not show small size aneurysms and a false negative result of 33% for PCOM aneurysm by MRA was reported previously.^5^ Therefore, in presence of high clinical suspicion to intracranial aneurysm as a cause of ptosis, brain CTA may be useful.

This case highlights that even though ptosis is a common side effect of botulinum injection in periocular muscles, other life threatening causes should also be considered especially when ptosis accompanied with severe aggravating pain. A complete history including exact time of beginning, the course and presence of other associated symptoms with ptosis, as well as a complete ocular examination with particular attention to extraocular muscles movements and pupil size could help to properly manage a new onset ptosis.

## Notes:

**How to cite this article:** Jamshidian-Tehrani M, Mehrjardi HZ, Kasaee A, Yadegari S. Ptosis following botulinum toxin injection in hemifacial spasm. Iran J Neurol 2019; 18(4): 184-6.
